# Identification of new p53 target microRNAs by bioinformatics and functional analysis

**DOI:** 10.1186/1471-2407-13-552

**Published:** 2013-11-21

**Authors:** Alessandra Bisio, Veronica De Sanctis, Valerio Del Vescovo, Michela A Denti, Anil G Jegga, Alberto Inga, Yari Ciribilli

**Affiliations:** 1 Laboratory of Transcriptional Networks, Center for Integrative Biology, CIBIO, University of Trento, Trento, Italy; 2 Laboratory of RNA Biology and Biotechnology, Center for Integrative Biology, CIBIO, University of Trento, Trento, Italy; 3 Division of Biomedical Informatics, Cincinnati Children’s Hospital Medical Center, Cincinnati, OH, USA; 4 Present address: LabSSAH, CIBIO-NGS facility, University of Trento, Trento, Italy

**Keywords:** p53 family, miRNA, Promoter occupancy, Transcription regulation

## Abstract

**Background:**

The tumor suppressor p53 is a sequence-specific transcription factor that regulates an extensive network of coding genes, long non-coding RNAs and microRNAs, that establish intricate gene regulatory circuits influencing many cellular responses beyond the prototypical control of cell cycle, apoptosis and DNA repair.

**Methods:**

Using bioinformatic approaches, we identified an additional group of candidate microRNAs (miRs) under direct p53 transcriptional control. To validate p53 family-mediated responsiveness of the newly predicted target miRs we first evaluated the potential for wild type p53, p63β and p73β to transactivate from p53 response elements (REs) mapped in the miR promoters, using an established yeast-based assay.

**Results:**

The REs found in miR-10b, -23b, -106a, -151a, -191, -198, -202, -221, -320, -1204, -1206 promoters were responsive to p53 and 8 of them were also responsive to p63β or p73β. The potential for germline p53 mutations to drive transactivation at selected miR-associated REs was also examined. Chromatin Immuno-Precipitation (ChIP) assays conducted in doxorubicin-treated MCF7 cells and HCT116 p53^+/+^ revealed moderate induction of p53 occupancy at the miR-202, -1204, -1206, -10b RE-containing sites, while weak occupancy was observed for the miR-23b-associated RE only in MCF7 cells. RT-qPCR analyses cells showed modest doxorubicin- and/or Nutlin-dependent induction of the levels of mature miR-10b, -23b, -151a in HCT116 p53^+/+^ and MCF7 cells. The long noncoding RNA PVT1 comprising miR-1204 and −1206 was weakly induced only in HCT116 p53^+/+^ cells, but the mature miRs were not detected. miR-202 expression was not influenced by p53-activating stimuli in our cell systems.

**Conclusions:**

Our study reveals additional miRs, particularly miR-10b and miR-151a, that could be directly regulated by the p53-family of transcription factors and contribute to the tuning of p53-induced responses.

## Background

The master regulator p53 is a prominent tumor suppressor gene, functioning in the cell as a tetrameric (dimer of dimers) sequence-specific transcription factor, able to bind to two copies of a decameric sequence with the RRRCWWGYYY consensus (where R stands for a purine, W for A/T and Y for a pyrimidine) representing the so called p53-response element (p53-RE) [1]. p53 is known to be inducible in response to a large number of cellular stress signals that, besides genotoxic stress, include carbon and oxygen deficiencies, perturbations of the translation apparatus, excessive proliferation signals, alteration in microtubule dynamics [2,3]. There are >100 established p53 targets genes that link p53 to cell cycle arrest, apoptosis, DNA repair and inhibition of angiogenesis [3-6]. More recently, p53 was demonstrated to modulate the expression of genes able to modify glucose as well as lipid metabolism, induction of autophagy, immune responses and cell motility [7-11].

A direct role of p53 on the activation of microRNA (miRs) expression as well as a role on selective maturation of microRNA precursors has been recently established [12,13]. miRs are small non coding RNAs typically of 21–25 nucleotides in length that regulate gene expression by inhibiting translation or repressing stability of target messenger RNAs including those coding for oncogenes and tumor suppressor proteins [14]. Dysregulation in miR expression has been reported in various cancers and can contribute to tumorigenesis [15]. The first evidence of a p53-dependent regulation of miR genes was provided by He et al., who identified a family of miRs, namely miR-34a-c, whose expression reflected the p53 status [16]. The authors demonstrated that genes encoding miR-34 family cluster were direct transcriptional targets of p53 and that their induced expression levels upon genotoxic or oncogenic stress was dependent on p53 expression, both *in vitro* and *in vivo*. Moreover, He et al. identified the DNA sequences responsible for the p53 responsiveness of those miRs. A year later another group of miRs, (miR-192, -194 and −215) was identified as targets of p53 and their ability to increase the level of CDKN1A (p21^CIP1^) and to function as drivers of cell cycle arrest was established [17]. Examples of feedback loops or regulatory circuits comprising p53, a target miR and target mRNAs were discovered. For example, p53-directed repression of c-Myc has also been linked to p53-dependent induction of miR-145 [18]. miR-107 was demonstrated to be activated by p53 and to cooperate in its cancer suppressive function through the inhibition of HIF-1β and, consequently, tumor angiogenesis [19]. The p53 targeted miR-34a was shown to modulate SIRT1 [20]. More recently, Jin et al. surprisingly found that p53 directly induced the transcription of miR-149*, which in turn can target the glycogen synthase kinase-3α mRNA, resulting in elevated expression of Mcl-1 and resistance to apoptosis in melanoma cells, thus providing a rational explanation for the poor ability of p53 to suppress melanoma progression [21].

Furthermore, it has been demonstrated that p53 itself can be indirectly activated by the miR-29 family members (miR-29a, -29b and -29c), which inhibit the expression of p85 alpha (the regulatory subunit of the phosphatidylinositol-3 kinase, PI3K) and CDC42 (Cell division cycle 42, a Rho family GTPase), thereby decreasing their inhibitory effect on p53 [22]. Alternatively, miRs can also negatively regulate p53 expression as observed for miR-1285, miR-504, miR-33, miR-380, miR-30d, miR-25 and miR-125b [23-28].

The mechanisms regulating *in vivo* p53 transactivation specificity still need to be fully understood, but require in most cases the interaction of p53 with its response element sequences (REs) at target promoters [4]. Recent evidences, including our studies using functional as well as DNA binding assays in yeast or mammalian cells or with cell extracts, demonstrated that maximal transactivation potential requires adjacent dimer binding sites [29-34]. A spacer between dimer sites even of 1 or 2 nucleotides conferred a negative impact, particularly for the p53-related protein p73 [31,35,36]. We also established that p53 can stimulate transcription, albeit at a reduced levels, from noncanonical response elements (half-sites and ¾ sites) [31], that do not provide for a p53 tetramer binding site. The same sequence-specific requirements that were shown to maximize the transactivation potential from full-site REs, appeared to be valid for the half-site REs [31]. This information is relevant to optimize pattern-based motif searches aiming at identifying functional p53 response elements within genomes [37-39].

In this study we used a regression-based predictor for p53 transactivation [39], to identify additional p53-target miRs through the presence of functional p53-REs in their promoter regions or in promoter regions of long noncoding RNA that are precursors of those miRs. We then used a yeast-based functional assay to determine the relative transactivation capacity of p53 family proteins towards the identified REs and Chromatin Immuno-Precipitation (ChIP) assays in human cells to investigate genotoxic-stress dependent p53 occupancy at the chromosomal sites containing those REs. Changes in the expression levels for mature miRs or precursors were measured by real-time qPCR using cell lines and treatments probing the direct involvement of p53. We propose miR-10b, -23b and -151a to be included in the list of direct p53 target miRs contributing to the fine-tuning of p53-induced responses.

## Methods

### Yeast reporter strains and media

We constructed a panel of 16 reporter strains in the budding yeast *Saccharomyces cerevisiae* containing the *Firefly* luciferase gene under the control of putative p53-REs (see Table 1 for the RE sequence and location with respect to miR genomic coordinates) predicted to control the expression of miR -10b, -23b, -34a, -106a, -145, -151a, -191, -198, -202, -221, -320, -328, -455, -671, -1204, -1206. To this aim we took advantage of the methodology of the well-established *delitto perfetto* approach for *in vivo* mutagenesis using oligonucleotides [40] starting with the master reporter strain yLFM-ICORE [41]. The strain contains the luciferase cDNA integrated at the chromosome XV downstream a minimal promoter derived from the *CYC1* gene. The ICORE cassette is located 5′ to the minimal promoter and enables high efficiency targeting of the locus by oligonucleotides that contain desired RE sequences. The targeting events were followed by phenotypic selection and clones examined by colony PCR and direct DNA sequencing (BMR Genomics, Padua, Italy).

**Table 1:** List of predicted p53-REs mapped nearby miR genes

**miR name**	**p53-RE Genomic location**	**p53-RE distance miRNA**	**Overlapping transcript**	**Distance from sense transcript TSS**	**p53- RE organization**		
					**DIMER 1**	**Spacer**	**DIMER 2**
**Consensus**	**-**	**-**	**-**	**-**	**RRRCATGYYY**	**N = 0 - 13**	**RRRCATGYYY**
miR-34a	Chr 1 - 9242203	- 30,476 bp	Intergenic	+ 123 bp	GGGCTTGCCT	-	GGGCTTGTTC
miR-10b	Chr 2 - 177013750	- 1,281 bp	HOXD3	+ 12,065 bp	TAACTCGTTG	GCTTTGACCTGTCT	GAACAAGTCG
miR-23b	Chr 9 - 97818687	- 28,803 bp	C9orf3	+ 135 bp	AGGTCAGTCA	TG	GGACATGTCC
miR-106a	Chr X - 133309906	- 5,678 bp	Intergenic	-	GTTATGTTC	ATGTGCTCAT	GTGCATGCCC
miR-145	Chr 5 - 148786372	- 23,837 bp	Intergenic	+ 68 bp	GCACCCGCCT	CGCCCCAATACG	GGGCTTGCCT
miR-151a	Chr 8 - 141734774	- 2,349 bp	PTK2	- 75,874 bp	TGGCTTGTTT	-	TGGCAAGTTC
miR-191	Chr 3 - 49063594	- 5,543 bp	DARLD3	+ 5,103 bp	GACCTTGTCT	TGCTTCC	AGCCATGTCA
miR-198	Chr 3 - 120112741	+ 1,774 bp	FSTL1	- 57,359 bp	AGGCAAGCTT	-	CAACAAGCCG
miR-202	Chr 10 - 135058647	+ 2,368 bp	RP13-49115.5	- 2,748 bp	GGGCATGTCT	-	TGGCAAGCCT
miR-221	Chr X - 45599946	+ 5,639 bp	Intergenic	-	GAACATGCAT	-	GCACATGTTT
miR-455	Chr 9 - 116880600	- 91,114 bp	COL27A1	- 49,395 bp	CTTCCTGCAT	AAGGCTTGGCGG	GCGCAAGCCC
miR-320a	Chr 8 - 22095461	+ 7,014 bp	Intergenic	-	AGGCATGGTG	-	CGGCATGCCT
miR-328	Chr 16 - 67331546	- 95,322 bp	ELMO3	+ 98,475 bp	CGGCAAGTCC	C	CAGCCAGTTC
miR-671	Chr 7 - 150896632	- 38,875 bp	CHPF2	- 32,953 bp	GGTCCAGCCC	TCTGGCCCCC	CAACAAGTCT
miR-1204	Chr 8 - 128808017	- 191 bp	PVT1	+ 1,238 bp	CGACAAGTTG	-	AGACTTGTTC
miR-1206	Chr 8 - 129002467	- 18,677 bp	PVT1	+ 1,037 bp	GGGCTAGTGA	-	AGGCATGTCT

miR name, genomic location of the predicted p53-RE (including chromosome number and genomic coordinates according to BLAT tool in UCSC Genome Browser, http://genome.ucsc.edu/), presence and distance from an overlapping primary transcript, p53 RE sequence and position relative to the miR sequence (obtained through the miRBase tool, http://www.mirbase.org/) are presented. p53-RE organization is shown grouping nucleotides in Dimer 1, Spacer (if any) and Dimer 2, according to the p53 consensus (in bold).

Yeast cells were grown in 1% yeast extract, 2% peptone, 2% dextrose with the addition of 200 mg/L adenine (YPDA medium). Selective minimal plates lacking tryptophan or leucine but containing adenine (200 mg/L) and dextrose as carbon sources were used (transformation in yeast and luciferase-based assay).

### Yeast expression vectors

For the expression of p53 family protein in yeast we used available CEN/ARS expression vectors (pTSG- or pLSG-based) harbouring alternatively the cDNA wild-type of p53, p63 or p73 under the control of the *GAL1,10* inducible promoter [31]. Among the various isoforms of p53 family members, we selected the full-length wild type p53 and the TA-p63β and TA-p73β isoforms as they showed the maximal transactivation potential in our experimental settings. The expression levels were modulated by the concentration of galactose in the culture medium (0.008%, 0.128% or 1%). The whole panel of 104 p53 germline missense mutants from the IARC R11 database (http://p53.iarc.fr) cloned in the pLS-Ad vector (providing for a constitutive expression of p53 mutants) [42] were used to test transactivation capability towards the miR-34a p53 RE. The pRS-314 or pRS-315 empty vectors were included as controls; these vectors contain respectively the TRP1 (as pTSG-) or LEU2 (as pLSG-) yeast selectable markers.

### Luciferase assays in yeast

The p53 family responsiveness of miRNA-associated REs was examined by transforming the panel of yLFM-RE strains with the p53 expression vectors. Transformants were obtained using the LiAc method and selected on minimal plates lacking tryptophan or leucine but containing adenine (200 mg/L) and dextrose as carbon source to allow respectively the growth of white colonies of normal shape and to keep the expression of p53 family members inhibited. After 3 days of growth at 30°C, transformants were streaked onto the same plates and allowed to grow for an additional day. For each reporter strain, the basal luciferase activity was measured from pRS314- or pRS315-transformants and used to calculate the fold of induction due to the expression of each p53 family member. For the luciferase assay we exploited the recently developed miniaturized yeast assay [43]. Transformant colonies were grown in 100 μl of the selective medium with 2% raffinose as carbon source (raffinose result in basal level of expression from the *GAL1,10* promoter), supplemented with different concentrations of galactose (0.008% and 0.128% for p53; 0.008% and 1% for the other members of the family, to obtain a low, moderate or high expression of the proteins) in a transparent 96-well plate and incubated under 150 rpm shaking for 16–24 hours at 30°C. OD_600_ was measured directly in the multi-well plate to normalize for cell density using a multilabel plate reader (Infinite M200-Pro, Tecan, Milan, Italy). 10 μl of cells suspensions were transferred to a white 384-well plate (Brand, Milan Italy) and mixed with an equal volume of PLB buffer 2X (Passive Lysis Buffer, Promega, Milan, Italy). After 15 minutes of shaking at room temperature, 10 μl of *Firefly* luciferase substrate (Bright Glo Luciferase Reporter Assay, Promega) were added. Luciferase activity was measured at the plate reader, using a luminescence program with 1” integration time.

### Cell lines and treatments

The human breast adenocarcinoma-derived MCF7 cell line was obtained from the InterLab Cell Line Collection bank, ICLC (Genoa, Italy). The colon adenocarcinoma HCT116 (p53^+/+^) cell line and its p53^−/−^ derivative were obtained from B. Vogelstein’s group (The Johns Hopkins Kimmel Cancer Center, Baltimore, Maryland, USA). Cells were normally maintained in DMEM or RPMI supplemented with 10% FCS and antibiotics (100 units/ml penicillin plus 100 mg/ml streptomycin).

Cells were treated for 24 hours with 1.5 μM doxorubicin (DXR) or 10 μM Nutlin-3A for p53 stabilization/activation. Stock solutions of Nutlin-3A were dissolved in 100% DMSO (stock solution 10 mM); DXR was dissolved in H_2_O (stock 10 mM). DXR was bought from Sigma-Aldrich® (Milan, Italy) whereas Nutlin-3A from Alexis® Biochemicals (Enzo Life Sciences, Exeter, UK). All the treatments were performed when cells reached 70-80% of confluence.

### RNA extraction and RT-qPCR

RNA extraction from human cell lines was done with Trizol reagent (InVitrogen, Life Technologies, Milan, Italy) following the manufacturer’s protocol. Cells were treated with 1.5 μM DXR or 10 μM Nutlin-3A for 24 hours prior to Trizol extraction. Quantification of mature microRNA expression was carried out using TaqMan MicroRNA Assay kits according to the manufacturer’s protocol (Applied Biosystems, Life Technologies), as described in Barbareschi et al. [44]. Specifically, ready-made TaqMan MicroRNA Assays (containing microRNA-specific forward and reverse PCR primers and microRNA-specific Taqman MGB probe) were used for the investigation of miR-34a (ABI P/N 000426), miR-10b (ABI P/N 000388), miR-23b (ABI P/N 000400), miR-151a (ABI P/N 002254), and miR-202 (ABI P/N 002363). We also quantified the U6 small nuclear RNA (RNU6B) (ABI P/N 4373381) as an endogenous control to normalize the levels of target microRNA. Complementary DNA was generated using the Taqman MicroRNA Reverse Transcription (RT) Kit (ABI P/N 4366596) according to the manufacturer’s instructions. Reverse transcriptase reactions contained 10 ng of total RNA as the template, 3 μl of gene specific stem-loop RT primer, 1.5 μl of 10X RT buffer, 0.15 μl of 100 mM dNTPs, 1 μl of MultiScribe reverse transcriptase, and nuclease-free water. The 15 μl reactions were incubated on a GeneAmp PCR System (Bio-Rad, Hercules, CA) for 30 minutes at 16°C, 30 minutes at 42°C, 5 minutes at 85°C, and then kept at 4°C. Quantitative RT-PCR was carried out using the Rotorgene 6000 (Corbett Life Science, Ancona, Italy) in 20 μl PCR reactions containing 1.33 μl of RT product, 10 μl of FastStart TaqManProbe Master (Roche, Milan, Italy, P/N04673417001), 7.67 μl of nuclease–free water, and 1 μl of MicroRNA Assay (Applied Biosystems, Life Technologies) buffer. Reactions were incubated at 95°C for 10 minutes, followed by 40 cycles of incubation at 95°C for 15 seconds and at 60°C for 1 minute. The quantification of protein coding mRNAs was performed using a Sybr green RT-qPCR approach. Total RNAs extracted with Trizol were converted using the RevertAid™ First Strand cDNA Synthesis Kit containing the M-MuLV Reverse Transcriptase following the manufacture’s recommendation (Thermo Scientific Inc., M-Medical, Milan, Italy). qPCR were carried out using the KAPA Sybr Green PCR mix (Kapa Biosystems, Resnova, Rome, Italy) with 12.5 ng of cDNA on the CFX384 real-time PCR detection system (BioRad, Milan, Italy). Primers were picked using the Primer-BLAST online tool (http://www.ncbi.nlm.nih.gov/tools/primer-blast/); sequences are available upon request. The Glyceraldehyde 3-phosphate dehydrogenase (GAPDH) and β2-microgobulin (B2M) were used as reference genes for normalization.

In all the qPCR assays, the threshold cycle data (C_t_) and baselines were determined using auto settings. The C_t_ value was defined as the fractional cycle number at which the fluorescence passed a fixed threshold. Fold changes were calculated using the comparative Ct method (ΔΔCt).

### Western blot analysis

To evaluate p53, p63 or p73 protein levels in yeast we cultured transformant colonies for 24 hours using selective medium containing 0.128% (p53) or 1% (p63β and p73β) galactose to induce the expression. Yeast cells were harvested, washed in ddH_2_O and lysed mechanically with glass beads as previously described [45]. 15 μg (p53) and 75 μg (p63β and p73β) were loaded on a 7.5% Acrylamide gel and separated by SDS-PAGE. DO-1, 4A4 (Santa Cruz Biotechnology, Milan, Italy) and ER-15 (Calbiochem, Merck, Millipore, Milan, Italy) antibodies were used for p53, p63 and p73 immunodetection, respectively. PhosphoGlycerate Kinase 1 (PGK1, detected by Ab clone number 22C5D8, InVitrogen, Life Technologies) was used as loading control.

To demonstrate p53 stabilization and activation upon treatment with doxorubicin or Nutlin-3A, MCF7, HCT p53^+/+^ and HCT p53^−/−^ cells were harvested 16–18 hours after the treatments and lysed using RIPA (Radio Immuno Precipitation Assay) buffer (150 mM sodium chloride; 1.0% NP-40; 0.5% sodium deoxycholate; 0.1% SDS, sodium dodecyl sulphate; 50 mM Tris, pH 8.0) supplemented with Protease Inhibitors cocktail (Roche, Milan, Italy) [46]. 50 μg of the soluble extracts were loaded on a 12% Acrylamide gel and separated by SDS-PAGE. p53 and p21 endogenous protein levels were detected with incubation with monoclonal antibodies (DO-1 and C-19 clones against p53 and p21 respectively, Santa Cruz Biotechnology). Glyceraldehyde 3-phosphate dehydrogenase protein (GAPDH, Ab clone 6C5, Santa Cruz Biotechnology) served as loading control. All antibodies were diluted in 1% non-fat skim milk dissolved in PBS-0.1% Tween20.

### Chromatin immunoprecipitation (ChIP) analysis

HCT116 p53^+/+^ and HCT116 p53^−/−^ or MCF7 cells were grown on 150-mm dishes and treated with 1.5 μM doxorubicin for 24 hours. Proteins were cross-linked with DNA by addition of 1% formaldehyde. After 10 minutes incubation at room temperature the reaction was stopped by addition of glycine at a final concentration of 0.125 M followed by additional incubation for 5 minutes. Cells were washed twice with 10 ml cold PBS, harvested in 1 ml PBS plus protease inhibitors (PI) (Complete EDTA-free, Roche), and lysed using an SDS lysis buffer (1% SDS, 50 mM Tris–HCl pH 8, 20 mM EDTA, 0.1 mg/mL Sonicated Salmon sperm DNA, 1X PI). In order to eliminate soluble p53 protein, lysates were incubated with gently shaking for 10 min and insoluble material was collected by centrifugation at 800 g at 4°C for 5 min. Pellets were resuspended in 0.5 ml of sonication buffer containing 0.25% SDS, 200 mM NaCl, 100 mg/ml of sonicated salmon sperm DNA and protease inhibitors and were sonicated to shear DNA to lengths between 150 and 500 base pairs (bp) using a Misonix S-4000 sonicator with a plate horn (Misonix, Newtown, Connecticut, USA). After 10-fold dilution in ChIP dilution buffer (16.7 mM Tris, 0.01% SDS, 1.1% Triton X-100, 1.2 mM EDTA, 167 mM NaCl), IPs were carried out overnight at 4°C with 2 μg of anti-p53 (DO-1, Santa Cruz Biotechnology) or 2 μg of normal IgG as a negative control. Fifty microliters of Dynabeads protein G magnetic beads (Invitrogen, Life Technologies) were added to each sample for 2–3 h, and the beads were then washed as indicated in the Upstate Biotechnology ChIP protocol. DNA was eluted firstly with 100 μL of TE with 1% SDS for 10 min at 65°C and a second time with 150 μL of TE with 0.67% SDS for an additional 10 min at 65°C. The cross-links were reversed overnight at 65°C. RNase A was added and incubated at 37°C for 30 min and then Proteinase K for 2 h at 56°C. DNA was then purified by QIAquick PCR purification KIT columns (Qiagen, Milan, Italy). Immunoprecipitated DNA was analyzed for p53 occupancy on selected chromosomal sites surrounding the predicted miR-associated p53 REs by RealTime-qPCR and fold enrichment of p53 binding to the respective DNA sequences was calculated by the comparative C_t_ method as described previously [47]. RealTime-qPCR was carried out with the KAPA SYBR Green PCR mix (Kapa Biosystems, Resnova) and all primers were checked for equal amplification efficiency. All PCR results were normalized to input controls. Three different DNA loci were used as ChIP negative controls (the CCNB1 exon 9, the Actin and GAPDH promoter). Sequences of all ChIP primers are available upon request.

## Results and discussion

### Identification of functional p53 response elements in miR genes

**Figure 1:**
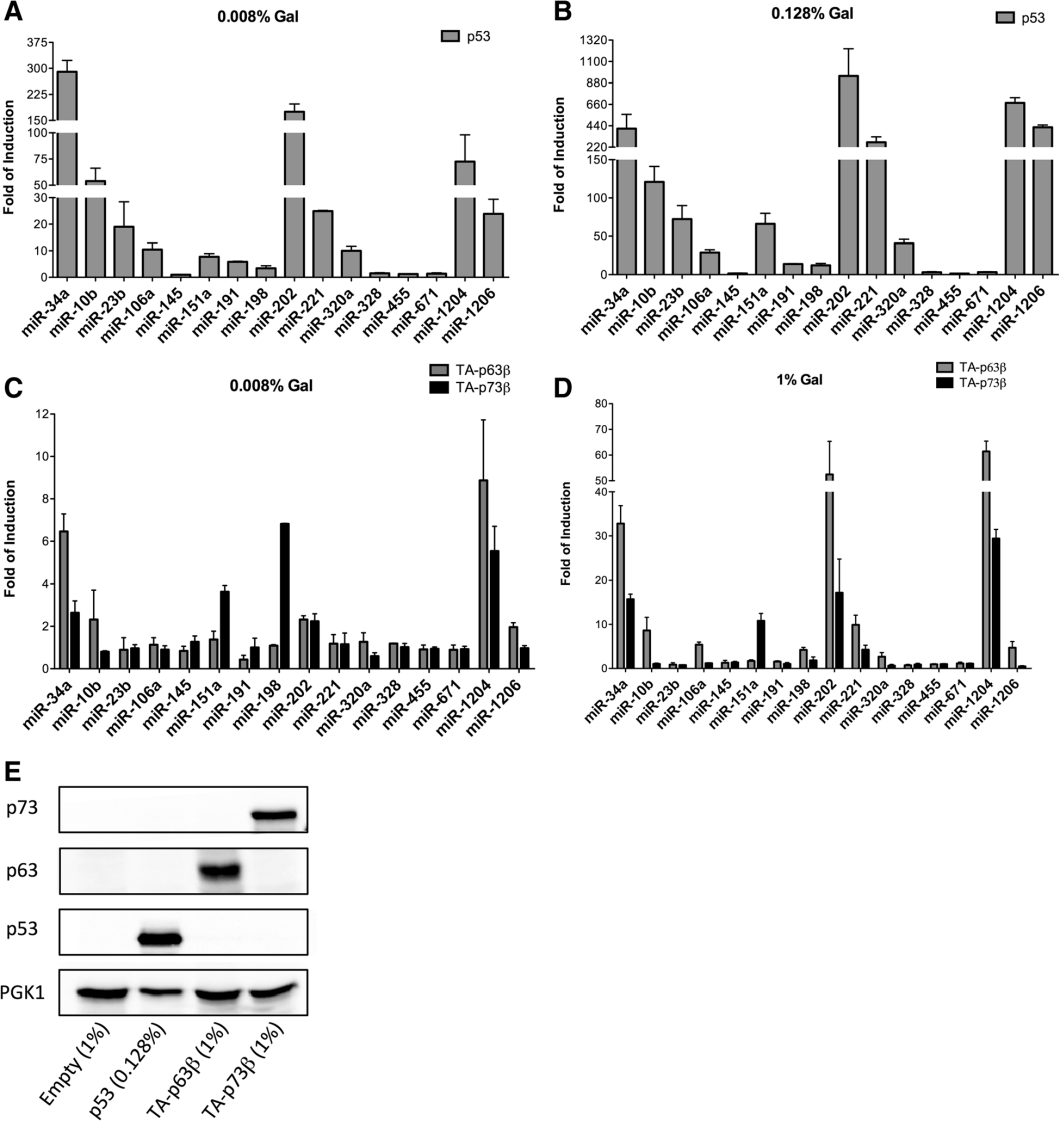
**p53 family members can transactivate p53-REs found in miR-associated promoter regions. A-B)** Transactivation potential of p53 protein tested on a panel of 15 putative p53-dependent miR-REs and miR-34a-RE (a positive control) using the yeast functional assay. The expression of p53 was modulated by increasing concentrations of galactose in the culture medium (**A**, 0.008%: a moderate p53 expression; **B**, 0.128%: high p53 expression). **C-D)** The same panel of p53 miR-REs was tested using the other members of the p53 family p63 and p73 (gray and black bars respectively). The expression of p63β and p73β isoforms was induced using two different concentrations of galactose (**C**, 0.008%: moderate expression; **D**, 1%: maximal induction). Results are presented as fold of induction calculated over an empty expression vector. Bars plot the averages and standard deviations of at least three independent biological repeats. **(E)** Western Blot establishing galactose-dependent expression of p53, p63 or p73 in yeast. PGK1 was used as reference.

We applied a predictor tool for p53 RE-transactivation potential [39] to identify candidate p53 REs within regulatory regions of miR genes or promoter regions of long noncoding RNAs containing pri-miR clusters. Based on this analysis several novel p53 target miRs can be predicted (Table 1). To confirm p53 responsiveness of the identified p53 REs we first applied a well-established quantitative reporter assay in the budding yeast *Saccharomyces cerevisiae*[37,43]. This assay was chosen as it provides a defined experimental system to measure transactivation potential of a panel of REs each cloned at the same chromosomal location in isogenic derivative reporter strains where wild type or mutant p53, as well as p53-related proteins p63 and p73, can be expressed, one at a time, from a inducible promoter [43]. 15 candidate miR-associated p53 REs were studied. The validated miR-34a p53 RE was included as a positive control. The results indicated that most of the identified p53-REs could be transactivated at low to moderate levels by wild type p53 (Figure 1A). As expected the responsiveness was proportional to p53 expression levels (Figure 1A and B). Based on results obtained both with low and high p53 expression, REs from miR-34a, -10b, -202, -1204 were highly responsive (HR), from miR-23b, -151a, -221, -320, -1206 were moderately-responsive (MR), from -106a, -191, -198 were weakly responsive. Putative REs from miR-145, -328, -455, -671 were not responsive to p53 in the yeast-based assay.

Next we tested p63 and p73 responsiveness of the same panel of REs, using the transactivation competent, TA-p63β and TA-p73β isoforms for these proof-of-principle experiments, as they exhibit higher relative transactivation potential compared to N-terminal truncated ΔN- and also compared to TA-p63α and TA-p73α isoforms [37]. As expected the transactivation potential of p63 β and p73β were much lower compared to p53 (Figure 1C and D). Only a subset of p53-responsive REs was active with p63 and p73 and included miR-34a, -202 and −1204 REs. Furthermore, differences in relative transactivation potential were observed in the comparison of the three family members. For example, miR-34a and −1204 were more responsive to p63 than to p73. Furthermore, we observed examples of selective lack of responsiveness: p73 towards miR-10b and −221 REs; p63 towards mir-151a. To verify the protein expression of the three p53 family members in yeast after galactose induction (0.128% and 1% galactose for p53 or p63 and p73, respectively, to match the experimental conditions used for the luciferase assays) we performed a western blot using antibodies specific for each transcription factor (Figure 1E).

**Figure 2:**
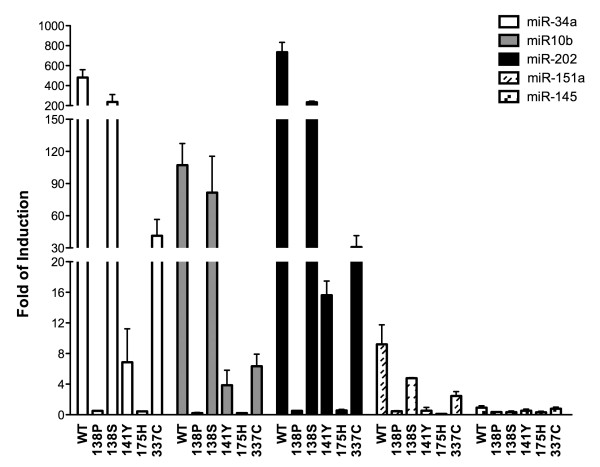
**p53-REs found in miR promoters can be used to classify p53 germline alleles associated with Li-Fraumeni Syndrome.** Five p53 missense mutants representative of partial function (A138S, C141Y, R337C) or loss-of-function (A138P, R175H) germline p53 alleles were tested for transactivation from 5 p53 miR-REs using the yeast functional assay. Yeast transformants were cultured over-night in selective medium and the luciferase activity was measured using the miniaturized assay format [43]. Bars represent the averages and standard deviations of at least three independent biological repeats.

For 5 REs, representative of strong, moderate, weak, and nearly-absent responsiveness to wild type p53, the functional assay was extended to five p53 missense germline mutations, of which three retain partial function (A138S, C141Y, R337C) and two are loss of function (A138P, R175H) [42]. Similar results were obtained with the responsive REs, confirming the functional classification of the p53 mutants examined (Figure 2); for example, A138S was near-wild type, while R337C was weakly active (<10% of wt activity) and 141Y almost inactive (<5% of wt activity). Given the important role of miR-34a as a positive modulator of p53-mediated responses [48,49] and our recent studies indicating that p53 mutant transactivation capacity can correlate with clinical variables in Li-Fraumeni patients [42,50], we decided to use the miR-34a reporter strain to examine the entire panel of 104 germline p53 alleles described in the R11 release of the p53-mutant IARC database [42]. The vast majority, 83 out of the total 104 alleles (79.8%) were considered loss of function (LOF, defined for the cases where a residual transactivation was lower than 25% of the wild type p53 activity). Eight (7.7%) retained a partial function phenotype (activity between 75% to 25% of wt p53), while 9 (8.7%) had the same transactivation potential as the wild-type. Interestingly, 4 alleles (R337H, H365Y, S366A, P82L) showed a transcriptional activity higher than wild-type p53 (>125%) and can be considered as super-transactivating alleles (ST). All the results are summarized in the Additional file 1: Table S1. As expected, while the vast majority of the LOF-p53 alleles hit the central portion of the p53 protein, associated with DNA binding capacity, the WT-, PT-, and ST-alleles were preferentially confined to the extremities (both N- or C-terminal domains). Compared to our previous classification based on four p53 REs derived from P21, MDM2, BAX and PUMA, 8 additional alleles are classified as LOF with miR-34a, while one (I305M) would change from LOF to partial function.

Overall these results identify a panel of p53 REs that based on the comparison with well-established REs from coding genes [31,37] suggest the potential for p53-mediated control of miR gene expression in vivo. Further, results suggested that miRs could be selectively or more specifically (e.g. -10b, -151a; -198) targeted by individual p53 family proteins, possibly contributing to the distinctiveness of the regulated networks and biological outcomes. Finally, the identified p53 miR-REs can be used to refine the functional classification of cancer associated p53 mutant alleles.

### p53-occupancy at endogenous miR-associated promoters in human cells

**Figure 3:**
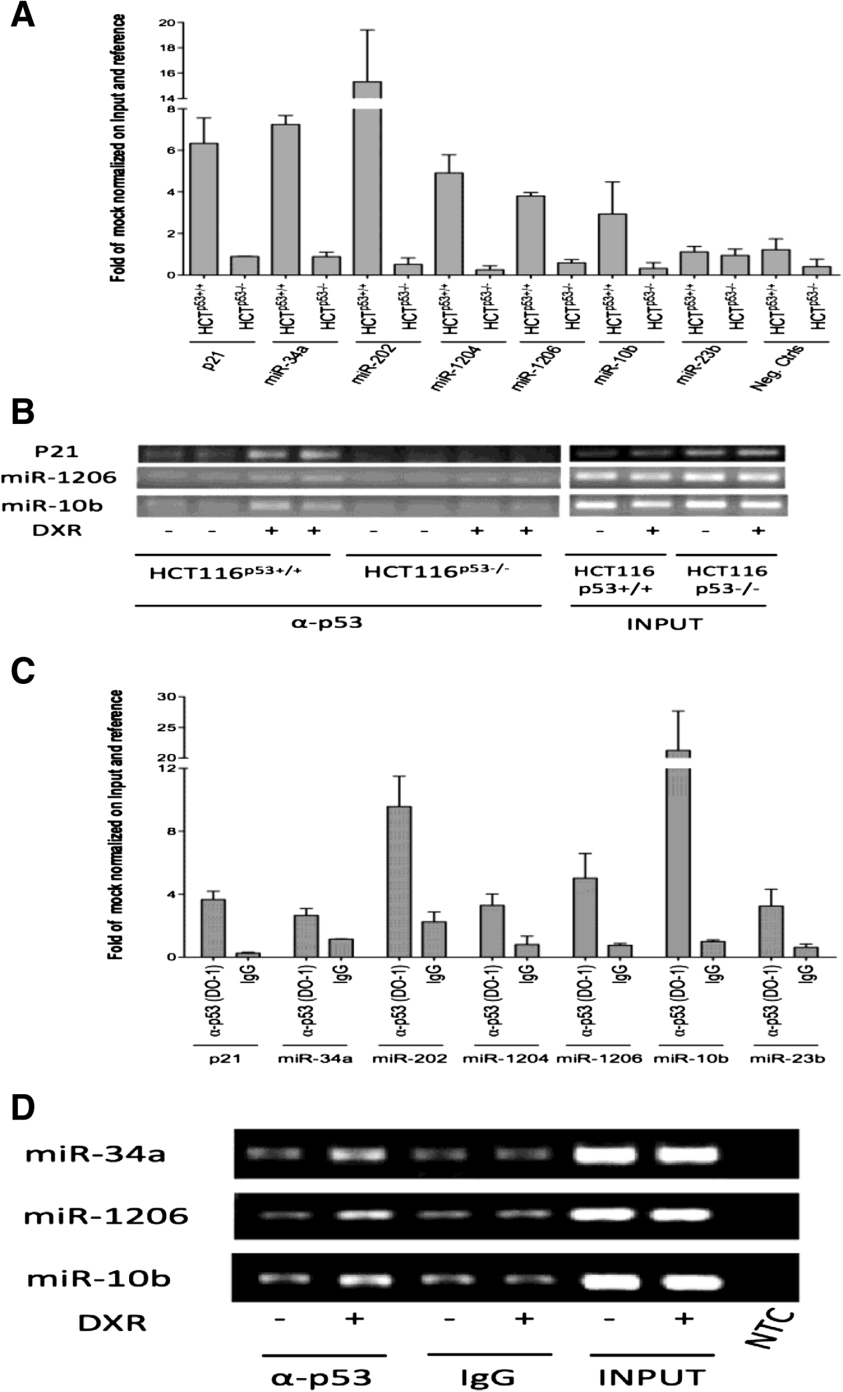
**p53 can bind chromatin region surrounding the identified p53 REs in miR genes. A)** ChIP assays were performed in HCT116 p53^+/+^(gray bars) and HCT116 p53^−/−^ (black bars) upon doxorubicin treatment for 24 hours. The results of Real-Time qPCR are presented as fold of mock treatment, normalized with respect to the signal obtained with Input DNA. The results from three control locations corresponding to promoter regions of Actin, GAPDH and exon 9 of CCNB1 genes were averaged and are also presented in panel **A** (Neg. Ctrls). P21 and miR-34a occupancy were measured as positive controls. Bars represent average and standard deviations of three independent experiments. **C)** ChIP assays of MCF7 cells treated with doxorubicin for 24 hours. Results obtained after ChIP with an antibody against IgG were included as a negative control for the p53 miR-REs. Examples of agarose gel analysis of standard ChIP-PCR are given in panels **B** and **D**. Specifically, panel **B** shows experiments performed in HCT116 p53^+/+^ and p53^−/−^ cells, while panel **D** presents results from MCF7 cells. The DO-1 p53 antibody was used for immunoprecipitation; NTC, no template control. Regions surrounding the established P21 and miR-34a p53 REs were examined as positive controls.

As the yeast functional data provided us information predominantly on the nature of the mapped p53-REs, specifically on the transactivation potential in an isogenic, ectopic context, we analyzed the correspondence of our results with the ability of p53 to physically interact with those sequences in their natural context in mammalian cells treated with a genotoxic agent (doxorubicin, DXR) known to result in p53 stabilization and activation [51,52]. To this aim we treated HCT116 p53^+/+^, HCT116 p53^−/−^ and MCF7 (p53 wt) cells with 1.5 μM DXR for 24 hours and performed ChIP assays. We selected all the miR promoters with REs classified as HR and some of the MR according to the p53 responsiveness in the yeast assay. In HCT116 p53^+/+^ cells, DXR-induced p53 occupancy was observed for all chromosomal coordinates surrounding miR-associated REs with the only exception of miR-23b. The relative increase in occupancy was comparable for miR-202 and miR-1204 sites to the well-established p53 target P21-5′ RE region and the p53-miR-34a target (Figure 3A and B). As expected in HCT116 p53^−/−^ cells we did not find any occupancy, confirming the specificity of the assay. The experiment was repeated in another p53 wild-type cell line, MCF7, using IgG as a control of IP specificity. Doxorubicin-induced occupancy was observed for all sites examined, including miR-23b. In particular, miR-202 and miR-10b promoters showed the highest relative induction of p53 occupancy (Figure 3C and D).

Downstream of and consistent with the yeast-based results, ChIP assays further supported the putative function of the identified p53 REs in modulating p53-mediated responsiveness of miR genes. However, the correlation between occupancy and transactivation is not direct, nor linear [4]. p63 and p73 occupancy was not investigated and awaits further studies to clarify the contribution of p53 family proteins on miR gene expression.

### Doxorubicin responsiveness of identified p53 target miRs in p53 wild type human cells

**Figure 4:**
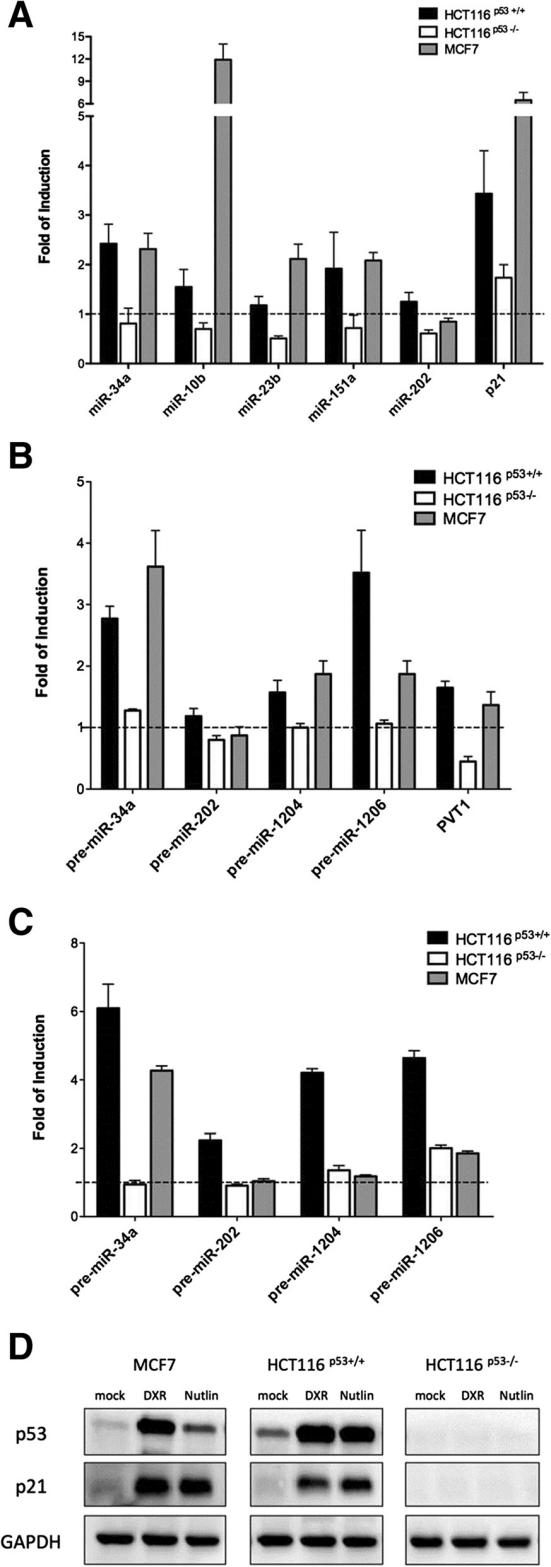
**p53-induced expression of mature and pre-miR genes.** RT-qPCR were performed in HCT116 p53^+/+^(black bars), HCT116 p53^−/−^ (white bars) and MCF7 (gray bars) cells upon doxorubicin **(A, B)** or Nutlin **(C)** for 24 hours. The expression of the processed mature miR **(A)** or of the pre-miR RNA **(B, C)** was tested. p21 mRNA expression was measured as p53-dependent positive control. PVT1 non-coding RNA expression levels were measured as additional evidence of p53-dependent expression of miR-1204 and miR-1206. Results are presented as fold of induction with respect to the mock condition. Bars plot average and standard deviations of three independent experiments. **(D)** Western Blot establishing stabilization of p53 protein in doxorubicin and Nutlin treated cells and the induction of the p53 target gene p21. GAPDH was used [6] as reference.

With the yeast-based assays we established the potential for p53-mediated transactivation of p53 REs associated with miR sites, while ChIP experiments established accessibility and potential recruitment of p53 at those sites. Next we examined if the expression levels of mature or precursor miR transcripts could be modulated by treatments resulting in p53 activation using again the HCT116 p53^+/+^, HCT116 p53^−/−^ and MCF7 cell line systems. The results indicated that of miR-10b, -151a and -23b are p53-responsive (Figure 4A). Consistent with ChIP analysis higher induction levels of mature miR-10b and -23b in response to DXR were observed in MCF7 than in HCT116 p53^+/+^ cells. The treatment did not result in miR induction in HCT116 p53^−/−^ cells, in fact some repression was apparent, particularly for miR-23b (Figure 4A). In contrast to RE transactivation potential and p53 occupancy studies, miR-202 expression did not change after the genotoxic treatment (Figure 4). Unfortunately, we were not able to measure miR-1204 or miR-1206 as the expression in these cells appeared to be below the detection limit of the qPCR in these cell lines (data not shown).

To exclude any impact of the miR maturation processes or low sensitivity of the mature miR assay systems, we also selected primers that can amplify the pre-miR RNA and performed RT-qPCR for miR-1204, miR-1206, miR-202 and miR-34a (Figure 4B). We also analyzed the expression of PVT1, the long non-coding RNA transcript comprising the miR-1204 cluster [53]. Weak, DXR-dependent induction was observed for PVT1, pre-miR-1204 and pre-miR-1206 in HCT116 p53^+/+^ and MCF7 cells. No changes were observed in HCT116 p53^−/−^ or repression of PVT1 (Figure 4B). To further confirm the direct involvement of p53 in the transcriptional regulation of those miRs we also treated the cells with the MDM2 specific inhibitor Nutlin-3A (Figure 4C). Except for pre-miR-34a, pre-miR-1204, -1206 and even −202 were responsive to Nutlin treatment only in the HCT116 p53^+/+^ cell line, highlighting cell type and treatment dependencies in the expression regulation. The effect of the treatments on p53 stabilization and activation was examined using western blot (Figure 4D).

miR expression analysis in doxorubicin treated cells differing for p53 status supported p53-mediated responsiveness for miR-10b, -151a and, limited to MCF7 cells, also -23b. The levels of induction were in general comparable to those of miR-34a. Despite the high transactivation potential of the associated p53 REs and the p53 occupancy analysis, the mature miR-202 was not responsive to p53-inducing treatment. This discrepant finding could be related to the relatively large distance between the mapped p53 REs and the pri-miR-202 transcript start site and/or to the inaccessibility of the site due chromatin structure. The p53 RE sequence does not fall within DNAse sensitive sites based on ENCODE data. We were not able to confirm the p53-dependent induction of mature miR-1204 and −1206 in our cell lines, although we detected weak induction of the long noncoding RNA containing the miR-1204 cluster and possibly evidence for an internal transcript comprising pre-miR-1206. A recent study established p53-dependent induction of Plasmacytoma Variant Translocation 1 gene PVT1 and miR-1204 [53] in HCT116 p53 wild type cells treated with doxorubicin. Our results confirm those findings and also suggest p53 recruitment internally to the PVT1 gene locus to possibly further modulate miR-1206 independently or in addition to the activation of the entire miR-1204-1208 cluster. Further studies are needed, including the use of cell lines expressing higher basal levels of PVT1 to examine whether miR-1206, and possibly −1207 and −1208 downstream, can be modulated by p53 family proteins also independently from PVT1 gene transcription. A link between p53 and modulation of miR-23b was also recently described and indirectly related to human papillomavirus (HPV)-mediated responses through inhibition of p53 function [54]. Our results further confirm miR-23b as a p53 target miR in other cancer–derived cell lines. A previous link between p53 and miR-151a, as well as FAK pre-mRNA that contains miR-151a, was proposed based on transient silencing of p53 in the hepatocellular carcinoma-derived HepG2 cells resulting in FAK and miR-151a up-regulation (Figure S4 in [55]). Our results in different cell models indicate instead the potential for positive modulation of this miR by doxorubicin treatment in p53 wild type cells. Bioinformatics-based predictions, transactivation potential of RE, occupancy and mature miR expression changes in doxorubicin treated cells, consistently indicate, to our knowledge for the first time, miR-10b as a p53 target gene.

### An expanded role of p53 in the modulation of microRNA expression

The study of the p53 gene transcriptional networks continues to raise particular interest in the field due to the increasing complexity of regulatory circuits and the functions of the extensive list of target genes spanning a myriad of different biological pathways. The discovery of p53-target miRs has led to the identification of several feedback and feed-forward loops that can lead to fine-tuning of p53-mediated responses. A few p53 target miRs, more prominently miR-34a, have been shown to act as bona-fide tumor suppressor genes [56,57]. Multiple evidence, comprising gene expression, ChIP-seq and phenotypic studies upon gene silencing or targeting in cell and animal models indicate a complex crosstalk between p53 and the related p63 and p73 proteins at the level of common and exclusive coding gene targets [58-61]. An integrated view of common and p53-family protein specific regulation of miR genes is however largely missing.

This work led to the identification of new p53 target miRs (miR-10b; -151a) and also confirmed or extended recent evidence from the literature (miR-1204, -1206; -23b). Proof-of-principle experiments also suggested miR genes worth of further analysis to ascertain a specific or selective role for p63 or p73 transcription in their expression. The weak p53-responsiveness towards p53 REs associated with miR-106a, -191, -198, -221 and −320 was not pursued in this study and awaits further investigation.

Perhaps surprising is the fact that the miR genes we propose (miR-10b; -151a) or confirm more in detail (miR-23b; -1204; -1206) as direct p53 targets do not fit intuitively with the expected p53-mediated functions. In fact all these miRs have been proposed to exhibit oncogenic activities or at least their over-expression has been correlated to aggressive cancer phenotypes in some tissues [55,62-66]. For example, the established potential for miR-10b to target both CDKN1A and CDKN2A mRNAs could in principle result in a p53-directed attenuation circuit of cell cycle arrest and senescence [62]. However, KLF4 mRNA has been described as a miR-10b target and KLF4 down-regulation in breast cancer cells has been reported to restore p53 functions leading to apoptosis [67]. Hence, in specific cellular contexts, it is possible that the p53-dependent regulation of miR-10b we discovered could result in a positive feedback loop stimulating p53 activity. Further, CpG islands upstream from the miR10b/10b* locus were found to be hyper-methylated in breast cancers and through ectopic expression an important role for miR-10b* in cell cycle inhibition was established [68].

It is known that miR functions can be highly context and tissue-dependent and their p53-mediated control in normal cells could potentially affect biological responses also not directly related to cell cycle control or apoptosis. For example, low levels of miR-23b resulting in higher levels of its target urokinase-type plasminogen activator could promote cervical cancer cell migration [54]. Finally, increasing evidence link p53 functions to innate and adaptive immunity and it could be speculated that miR-23b as well as PVT1 and the miR-1204 cluster regulation could be relevant in this context [53,69]. Interestingly, functional enrichment analyses of predicted targets of both miR-10b and -151a showed enrichment for neuron generation/development and brain-related phenotypes (uncorrected p-value < 0.05; data not shown).

## Conclusions

In our study, bioinformatics-based predictions, transactivation potential of putative p53 REs, p53 occupancy at the endogenous RE positions, and mature miR expression changes in cell lines differing for p53 status, were combined to identify miRs that are direct transcriptional targets of wild type p53. We established that miR-10b and miR-151a are new p53 target genes and also confirmed *cis-*mediated regulation by p53 of miR-1204, -1206 and -23b. Further studies are warranted to establish the biological implications of the newly identified p53 target miRs.

## Competing interests

The authors declare that they have no competing interests.

## Authors’ contributions

AB, AGJ, AI and YC designed research; AB, VDS, VDV, MAD, AGJ, AI and YC performed research; AB, VDS, MAD, AGJ, AI and YC analyzed data; AB, MAD, AGJ, AI and YC wrote the paper. All authors read and approved the final manuscript.

## Pre-publication history

The pre-publication history for this paper can be accessed here:

http://www.biomedcentral.com/1471-2407/13/552/prepub

## Supplementary Material

Additional file 1: Table S1 Transactivation potential of 104 Li-Fraumeni associated germline p53 missense mutations towards the miR-34a p53-RE. Click here for file
