# Supplementary material for: Identification of new p53 target microRNAs by bioinformatics and functional analysis
Source: BMC Cancer. 2013 Nov 21;13:552. doi: 10.1186/1471-2407-13-552 (PMC4225545; doi:10.1186/1471-2407-13-552)
Supplement: Additional file 1: Table S1 — Transactivation potential of 104 Li-Fraumeni associated germline p53 missense mutations towards the miR-34a p53-RE. [file 1471-2407-13-552-S1.pdf]

Table S1

p53 allele	transactivation % vs WT	SD	Score
empty	0,31	0,08	-
WT	100,00	14,96	WT
P82L	176,27	31,63	ST
V97I	44,73	12,44	PF
G105C	4,93	1,61	LOF
S106R	101,23	16,38	WT
Y126C	3,51	0,87	LOF
K132E	0,22	0,07	LOF
M133R	0,69	0,17	LOF
M133T	8,09	2,60	LOF
A138S	77,24	25,86	WT
C141Y	1,14	0,00	LOF
A138P	0,67	0,15	LOF
Q144L	44,74	9,87	PF
P151S	0,85	0,24	LOF
P151T	2,06	0,39	LOF
P152L	20,60	3,49	LOF
G154V	1,05	0,36	LOF
T155N	0,39	0,13	LOF
R156H	65,50	17,40	PF
R158G	2,70	0,26	LOF
R158H	1,70	0,15	LOF
I162N	1,00	0,30	LOF
Y163C	0,20	0,04	LOF
V167K	19,31	5,27	LOF
V172F	0,46	0,16	LOF
V173M	0,04	0,01	LOF
R174G	4,20	0,81	LOF
R175G	4,58	1,19	LOF
R175H	0,32	0,02	LOF
E180K	6,05	0,43	LOF
R181C	4,92	0,92	LOF
R181H	6,37	1,12	LOF
R181L	50,48	1,69	PF
R181P	0,60	0,11	LOF
A189V	89,27	8,94	WT
H193R	1,06	0,34	LOF
R196P	0,50	0,06	LOF
V197E	1,46	0,05	LOF

p53 allele	transactivation % vs WT	SD	Score
V197M	12,83	2,20	LOF
N210Y	75,27	13,08	WT
R213P	0,58	0,13	LOF
R213Q	2,18	0,53	LOF
P219S	18,37	5,11	LOF
Y220C	0,98	0,19	LOF
Y220S	1,24	0,12	LOF
S227T	100,42	9,99	WT
H233D	2,27	0,43	LOF
Y234C	5,41	0,28	LOF
N235D	9,24	1,13	LOF
N235S	11,03	0,33	LOF
Y236C	0,08	0,01	LOF
M237I	0,06	0,02	LOF
C238G	0,06	0,00	LOF
C238S	0,22	0,01	LOF
C238Y	0,25	0,06	LOF
S241F	0,27	0,02	LOF
S241T	0,29	0,02	LOF
C242Y	0,21	0,01	LOF
G244D	0,33	0,03	LOF
G244V	0,25	0,04	LOF
G245C	0,30	0,08	LOF
G245D	0,28	0,03	LOF
G245S	0,26	0,04	LOF
G245V	0,26	0,05	LOF
M246V	0,34	0,03	LOF
R248Q	0,25	0,05	LOF
R248W	0,18	0,02	LOF
I251M	11,81	1,89	LOF
T256I	19,53	2,38	LOF
L257Q	0,20	0,02	LOF
E258K	0,30	0,05	LOF
L265P	0,21	0,01	LOF
R267Q	8,81	1,02	LOF
E271V	15,59	3,38	LOF
Y272L	5,40	1,84	LOF
R273C	0,45	0,15	LOF
R273G	0,29	0,07	LOF

p53 allele	transactivation % vs WT	SD	Score
R273H	0,29	0,07	LOF
R273L	0,28	0,01	LOF
R273S	0,14	0,02	LOF
C275Y	0,11	0,03	LOF
C277Y	0,33	0,11	LOF
P278L	0,08	0,02	LOF
P278S	0,15	0,02	LOF
R280K	0,16	0,01	LOF
D281G	0,26	0,08	LOF
D281V	0,25	0,04	LOF
R282G	0,09	0,03	LOF
R282Q	7,16	0,17	LOF
R282W	1,55	0,18	LOF
R283C	48,91	9,31	PF
R283H	33,76	2,78	PF
E285Q	106,88	15,90	WT
E286A	10,43	3,20	LOF
R290H	75,68	5,17	WT
K292I	66,87	5,93	PF
G293W	109,91	18,96	WT
I305M	25,47	3,57	PF
R306P	19,69	1,92	LOF
G325V	85,67	5,03	WT
R337C	10,36	1,47	LOF
R337H	132,00	26,04	ST
L344P	0,16	0,03	LOF
H365Y	127,51	11,42	ST
S366A	139,86	23,12	ST

Score	number	percentage
ST	4	3,8
WT	9	8,7
PF	8	7,7
LOF	83	79,8
TOT	104	100

Score based on relative activity:
- LOF = <25%
- PF = 75%>x>25%
- WT = 125%>x>75%
- ST = >125%

The transactivation potential of Li-fraumeni associated p53 mutations can be assessed with miR-34a p53 responsive yeast strain. We employed our established yeast functional assay, using the miR-34a reporter strain, to examine the entire panel of 104 germline p53 alleles described in the R11 release of the p53-mutant IARC database (Monti et al., 2007). Yeast transformants were cultured in selective medium and the luciferase activity was measured after 24 hours using the miniaturized assay protocol (Andreotti et al., 2011). To rank the 104 p53 alleles, the transactivation potential was calculated as percentage of p53 wild-type (WT). p53 alleles were classified according to a score based on relative transactivation activity (TA). As schematized down on the left, we considered Loss of Function (LOF in the table, highlighted in orange) a TA below 25% compared to p53 WT; Partial Function (PF, green) when the TA was between 75% and 25%; a TA between 75% and 125% was evaluated as wild-type (WT, white); in case a p53 allele showed a TA even above 125%, it was scored as super-transactivating (ST, light blue). The table shows for each p53 allele the average of 4 indepenedent measurements, the standard deviation (SD) and the score. Empty vector was used as negative control (highlighted in yellow). We grouped all the p53 alleles in the panel down to the left according to the score and calculated the frequencies of each group of TA.

Monti P, Ciribilli Y, Jordan J, Menichini P, Umbach DM, Resnick MA, Luzzatto L, Inga A, Fronza G: **Transcriptional functionality of germ line p53 mutants influences cancer phenotype**. *Clin Cancer Res* 2007, **13**(13):3789-3795.

Andreotti V, Ciribilli Y, Monti P, Bisio A, Lion M, Jordan J, Fronza G, Menichini P, Resnick MA, Inga A: **p53 transactivation and the impact of mutations, cofactors and small molecules using a simplified yeast-based screening system**. *PLoS One* 2011, **6**(6):e20643.
